# Perspectives of substance use disorder counselors on the benefits and drawbacks of medications for opioid use disorder

**DOI:** 10.21203/rs.3.rs-4331201/v1

**Published:** 2024-05-03

**Authors:** Nicholas C. Cardamone, Rebecca E. Stewart, Kyle M. Kampman, Steven C. Marcus

**Affiliations:** University of Pennsylvania, Perelman School of Medicine; University of Pennsylvania, Perelman School of Medicine; University of Pennsylvania, Perelman School of Medicine; University of Pennsylvania, School of Social Policy and Practice

**Keywords:** Medications for opioid use disorder, opioid use disorder, substance use disorder counselors, stigma, MOUD

## Abstract

**Background:**

Medications for opioid use disorder (MOUD) are among the best tools we have to combat the opioid epidemic. Yet, use of MOUD among people with opioid use disorder (OUD) remains low. Interventions to increase MOUD access in the United States have largely focused on improving organizational capacity and addressing funding barriers, yet stigma toward MOUD may inhibit uptake even where MOUD is readily available. Substance use disorder (SUD) treatment counselors likely have considerable influence on a client’s choice to initiate and adhere to MOUD, but beliefs that counselors convey about MOUD in interaction with clients are understudied. The current study explores what advantages and disadvantages that SUD treatment counselors communicate about buprenorphine, methadone, and naltrexone.

**Methods:**

From June to December 2021, we surveyed counselors from publicly-funded SUD treatment agencies under a municipality-wide mandate to offer MOUD to all clients with OUD. Counselors were asked to describe, in a free-response format, the most important advantages and disadvantages to communicate to their clients about taking buprenorphine, methadone, and naltrexone. Counselor responses were coded for one or more advantage and disadvantage.

**Results:**

A total of 271 SUD counselors from 29 agencies completed the survey, generating 1,995 advantages and disadvantages across three types of MOUD. The most frequently reported advantage across all three types of MOUD was their ability to reduce cravings and illicit drug use. The most frequently reported disadvantage related to the potential for some types of MOUD to develop long-term medication dependence.

**Conclusions:**

As the availability and variety of MOUD treatment options continue to expand, it is important that SUD counselors are equipped with evidence-based recommendations for OUD care. We identified misalignments with the MOUD-prescribing evidence base and stigmatizing language toward MOUD within counselors’ responses, highlighting the potential to refine training materials for MOUD and mitigate stigmatizing beliefs.

## Background

Medications for opioid use disorder (MOUD) including buprenorphine, methadone, and naltrexone are effective at reducing both opioid related and all-cause mortality (1). Historically, the availability of MOUD has been limited by stringent regulations on agencies. However, with the state-by-state removal of prior authorization requirements, culminating in the January 2023 federal removal, MOUD treatment capacity has increased in recent years (2, 3) and a number of efforts to increase buprenorphine capacity have shown success in certain local and state contexts (4–7). As of 2020, an estimated 42% of the U.S. population live within a 10-mile radius of a treatment facility that offers the three main types of MOUD (8). Although more available than ever, less than a third of people with OUD or active opioid use reported receiving MOUD in the past year (9) and many individuals discontinue MOUD within a few weeks of starting (10).

Stereotypes and stigma pervade the OUD treatment system and are considerable barriers to treatment engagement, quality care, and long-term health outcomes for people with OUD (11). A form of stigma specific to the idea of using MOUD, called “intervention stigma” (12), has been documented across the OUD treatment continuum, including in reports from community pharmacists (13), counselors and peer recovery coaches in substance use disorder (SUD) treatment facilities (14, 15), SUD treatment agency directors (16, 17), and OUD patients themselves (18), MOUD stigma among counselors can present in different ways, such as via claims that MOUD encourages undesirable behaviors, is harmful to one’s physical or mental health, or is incompatible with “true” recovery (19).

MOUD stigma among counselors is concerning considering that, in outpatient settings, counselors have frequent touchpoints with clients, give advice and direction during client interactions, and help plan and connect them to MOUD treatment (20, 21). Counselors help people with OUD navigate recovery, yet there is little information about how they talk about MOUD during client interactions (12). To fill this gap, we surveyed counselors from SUD treatment agencies about how they communicate the advantages and disadvantages of each type of MOUD to their clients.

## Methods

### Procedures

From June to December 2021, we surveyed staff of treatment agencies that serve Medicaid recipients with SUD in the Philadelphia Metropolitan Area. These treatment agencies are reimbursed by Community Behavioral Health (CBH), the largest Medicaid payer for behavioral health in Philadelphia, and are mandated to provide MOUD to their clients with OUD (22). In collaboration with CBH, we solicited participation from executive directors of (n = 49) agencies in the CBH network to distribute the survey to counselors, therapists, or any clinician who provides psychosocial support to individuals with OUD. Counselors were provided informed consent and completed the survey instrument online via REDCap, a secure, HIPAA-compliant web-based application for data collection and storage (23). Respondents were sent a $25 Amazon gift card upon survey completion. Procedures were approved by the City of Philadelphia’s Institutional Review Board and all data were deidentified prior to analysis.

### Survey

Counselors were asked to describe, in a free-response format, the most important advantage and disadvantage they mention when discussing each of the three medications with their client. After completing the survey, counselors completed a demographic questionnaire (e.g. age, gender, ethnicity, education, years of experience, etc.). Agency directors provided information about the availability of each of the three medications at their agency.(17)

### Analysis

We employed thematic analysis methods (24) to code the advantages and disadvantages counselors reported for each medication. Two members of the authorship team (Author Initials, Author Initials) first collaboratively coded responses, resolving conflicts through discussion until satisfactory thematic saturation and agreement was reached. Missing responses (i.e. “I don’t know”, “not applicable”, “unintelligible”) were removed from the agreement analysis. The remaining responses were then independently coded and grouped, returning a kappa of .78 and .75 for advantages and disadvantages, respectively, indicating high inter-rater agreement (25).

## Results

### Sample Characteristics

A total of 271 counselors completed the survey from 29 (59.1% response rate) distinct agencies who serve Medicaid recipients in Philadelphia. Five counselors could not be linked to an agency. Respondents were mostly female, White, and had completed a bachelor’s degree (Table 1). The typical respondent had over a decade of professional experience and close to five years of experience at their current agency. Most respondents worked for agencies that dispensed or prescribed buprenorphine and naltrexone whereas only about half worked for agencies prescribe or dispensed methadone.

### Advantages.

There was a total of 1,055 reported advantages across the three types of MOUD from 250 respondents. Twenty-one counselors submitted blank responses and could not be coded for an advantage. There were 375 reported advantages from 245 respondents about buprenorphine, 335 advantages from 238 respondents about methadone, and 345 advantages from 234 respondents about naltrexone. Each advantage was coded into one of 28 themes (see Supplemental File 1).

Table 2 shows most the five most commonly reported advantages across the three types of MOUD: (1) reduces urges to use (42%), (2) reduces use of illegal opioids (37%), (3) flexibility (25%), (4) supports recovery lifestyle (20%), (5) reduces withdrawal symptoms (17%). Overall, the ability to reduce opioid cravings and use were the most frequently mentioned clinical benefits for all three types of MOUD. Other clinical effects mentioned include buprenorphine and methadone’s ability to reduce overdose risk and withdrawal symptoms, and naltrexone’s ability to block opioid receptors and prevent the sedative and euphoric effects associated of opioid use.

Approximately a third of respondents mention the ability for buprenorphine and methadone to reduce the use of illicit substances, jumpstart treatment readiness, and support of a recovery lifestyle. A smaller share of counselors (20%) associated these qualities with naltrexone. Instead, counselors described the advantages of naltrexone’s monthly dosing cycle: flexibility, convenience, and less interference with daily life.

### Disadvantages

There was a total of 940 reported disadvantages across the three types of MOUD from 245 respondents. Twenty-six counselors submitted blank responses and could not be coded for an advantage. There were 329 reported disadvantages from 239 respondents about buprenorphine, 353 disadvantages from 237 respondents about methadone, and 258 disadvantages from 218 respondents about naltrexone. Each disadvantage was coded into one of 26 themes (see Supplemental File 1).

Table 3 shows most the five most commonly reported disadvantages across the three types of MOUD: (1) it creates a long-term dependency (30%), (2) has harmful side effects (28%), (3) it is inconvenient (27%), (4) misuse potential (17%), (5) and difficult medication adherence (15%). Responses related to side effects frequently appeared for all three medications, including reports of cognitive and physical mal effects after taking buprenorphine or methadone and nausea or allergic reactions after a naltrexone administration. Notably, many counselors reported the tendency for buprenorphine and methadone to cause withdrawal symptoms which make it difficult for some clients to taper off.

The main disadvantages that counselors communicated about buprenorphine and methadone related their “addictive” properties. The words “reliance”, “dependence”, “habit”, or “crutch” frequently appeared in counselor responses when describing buprenorphine and methadone. Many counselors expressed concerns about buprenorphine or methadone developing physical dependencies and the difficulty involved with weaning off the medications. In addition, counselors often described the inconvenience, stigma, and psychological attachment involved with daily dosing at a methadone clinic. Responses which mention the methadone clinic often included phrases like, “time consuming”, “schedule”, and “commitment”.

Counselors mentioned misuse potential (incl. “abuse potential”, “selling”, “mixing with other substances”, “use to get high”) in 24% of buprenorphine responses, 18% of methadone responses, and 7% of naltrexone responses. Counselors characterized MOUD misuse as selling or diverting buprenorphine and mixing methadone with other substances to achieve euphoric effects. The disadvantages counselors offered for naltrexone were primarily related to its unique properties, including the requirement of abstinence before being induced and the possibility the blocker will fail or wear off before the next dose could be administered.

Stigma directed at the concept of using MOUD as an intervention for OUD were uncommon among respondents, but not absent. Phrases like “it’s substituting one drug for another” or “it’s still a drug” appeared in 18 (8%) responses for buprenorphine, 12 (5%) responses for methadone, and two (1%) responses for naltrexone.

## Discussion

It is unknown how substance use counselors or other non-prescribing staff present MOUD to their clients with OUD (26, 27). We surveyed counselors from Philadelphia’s publicly-funded behavioral health system about the advantages and disadvantages they convey about MOUD to their clients. In general, counselors reported that each type of MOUD has certain beneficial qualities related to their ability to reduce urges to use and promote recovery. However, counselors also noted drawbacks, such as buprenorphine’s misuse potential, the burden involved with having to be dosed methadone at a clinic, and the chance naltrexone may fail due to issues associated with its opioid blocker attribute.

A recent evidence synthesis from the National Institute of Drugs and Alcohol (NIDA) equates the effectiveness of methadone and buprenorphine at treating OUD in most treatment contexts (28). In line with this evidence, the counselors in our sample equally praise the effectiveness of the two agonists. Yet, many respondents also claimed that the disadvantages of buprenorphine and methadone is that they can be sold or used to “get high.” The diversion risk (i.e. using medication for anything other than its intended purpose) of opioid agonists continues to be under debate. There is little evidence to support that diverted buprenorphine is primarily used for euphoric effects – several U.S. found that people with OUD who misuse their prescribed buprenorphine reported motivations ranging from self-treatment, pain-relief, to withdrawal symptoms management (29, 30).

Few respondents mentioned the diversion potential and overdose risk of methadone. Previous research has indicated that most overdose deaths involving methadone have occurred in populations that use methadone for pain relief, not OUD treatment (31). Nonetheless, there has been a surge of scholarly interest and concern about the diversion risk of take-home methadone doses (31, 32). The emerging evidence indicates that overdoses involving methadone decreased (not increased) among people with OUD in the U.S. after the onset of the pandemic (33, 34).

A recent NIDA report notes that naltrexone has little-to-no diversion risk and, as expected, diversion was not commonly communicated as a disadvantage from our respondents (35). A number of respondents mentioned barriers associated with having to detox completely before being induced on naltrexone, echoing previous reports of patient experiences on extended-release naltrexone (36). In general, respondents had less to say about naltrexone. Naltrexone had the fewest number of codes extracted and substantially lower character counts than methadone and buprenorphine. This may be due to lack of familiarity and under-provision of naltrexone both locally and nationally (37). Approved by the U.S. Food and Drug Administration in 2010, naltrexone has a shorter history than buprenorphine and methadone. Some evidence suggests that naltrexone may be stigmatized less than buprenorphine and methadone due pharmacological and regulatory differences (19). Overall, our findings align with previous qualitative work with SUD counselors and supports the idea that MOUD stigma presents differently across MOUD types (20, 38).

Only a small number of respondents reported “explicit” forms of MOUD stigma (e.g. “it’s still a one drug for another”, “it’s still a drug”). However, a considerable share of responses contained language which implied that MOUD had addictive properties, harmful effects, or didn’t count as “clean time”. These misconceptions The DSM-V states that tolerance and withdrawal in the context of “appropriate medical treatment” is not considered criteria for substance use disorder (39). Indeed, physical dependence alone is not among the criteria for a SUD diagnosis, evidence of negative consequences associated with continued is also required. These misconceptions are worrying in light of recent work in our system which shows an association between agency MOUD utilization and the endorsement of stigmatizing beliefs toward MOUD by agency leadership (17).

Encouragingly, MOUD stigma can be acted on. A series of recent randomized controlled trials to promote MOUD uptake include components that address MOUD stigma, such as facilitation collaboration with community-based treatment settings (40), implementation coaches (41), and local change teams (42). The findings from our study suggest that there may be opportunities for counselor training interventions that specifically focus and standardize how to present medications to their clients with OUD. Our results also indicate the importance of tailoring destigmatization efforts to specific treatments (12) in order to provide people with OUD with all options for effective care. Future research should focus on shared decision-making models between counselors and clients and how they might be a lever to treatment success (43).

There are several limitations to the current investigation. Counselors self-reported how they discussed the advantages and disadvantages of medications so it is possible their responses were driven by social desirability, particularly in a system that has mandated the use of MOUD. Future investigations should corroborate these counselor reports with independently-coded recordings of counselor-client interactions. In addition, the availability and routine use of MOUD varies greatly across agencies. Counselors may have differential experiences with MOUD based on the medications their agencies provide and familiarity may lead to more perceived advantages. It is likely that counselors perceive different advantages and disadvantages about oral, sublingual, or injectable forms of buprenorphine which the study does not capture. Lastly, our sample is geographically limited to one large city; it is possible our findings do not generalize to other regions in the United States. Training requirements of substance use disorder counselors vary widely by state and Pennsylvania’s licensure requirements are among the most stringent for both undergraduate and graduate-level SUD counselors (44, 45). In addition, abstinence-oriented treatment facilities are more common in rural regions, limiting exposure to MOUD (20). Nonetheless, we expect that our results may help leaders to anticipate and prepare for stigma when MOUD is implemented in their agency.

## Conclusions

There have been dramatic changes to the OUD treatment landscape in the last decade and the term “MOUD” encompasses a variety of medications, each with their own pharmacological properties, drawbacks, and associated stigma. New treatment options, shifting post-pandemic treatment regulations, and changes to the street drug supply have added additional complexity. SUD treatment counselors play an important role in OUD treatment, but we show that the advantages and disadvantages counselors communicate vary across buprenorphine, methadone, and naltrexone and are inconsistent with the MOUD-prescribing evidence base. Our study highlights the need for ongoing education, training, and standardization around MOUD for non-prescribing SUD treatment professionals.

## Figures and Tables

**Table 1:** Characteristics of participating counselors

Characteristic	M (SD)
Age	43.5 (13.5)
Number of years of working experience	10.7 (9.7)
Number of years in current position	4.6 (5.7)
Gender:	% (N)
Female	192 (70.8%)
Male	73 (26.9%)
Not Reported	6 (2.2%)
Non-binary	0 (0%)
Education:	
Bachelor’s degree or above	238 (87.8%)
Master’s degree or above	165 (60.8%)
Race:	
Asian/Pacific Islander	7 (2.6%)
Black	100 (36.9%)
Native American	1 (0.4%)
White	139 (51.2%)
Other	18 (6.6%)
Not Reported	6 (2.2%)
Ethnicity	
Hispanic	19 (7.0%)
Not Hispanic	249 (91.9%)
Not Reported	3 (1.1%)
Agency dispenses or prescribes medication onsite	
Buprenorphine	197 (72.7%)
Methadone	128 (47.2%)
Naltrexone	222 (82.0%)
Unknown	5 (1.8%)

**Table 2:** Most prevalent advantages and illustrative excerpts

Advantages	Buprenorphine (245 responses)	n (%)	Methadone (238 responses)	n (%)	Naltrexone (234 responses)	n (%)	Total (250 responses)
**“No advantages”**	-	1 (0%)	-	7 (3%)	-	0 (0%)	7 (3%)
**Reduces urges to use**	*“Curbs cravings while preserving general functioning.”*	76 (31%)	*“Helps reduce cravings”*	60 (25%)	*“Helping with triggers and cravings.”*	57 (24%)	104 (42%)
**Reduce use of illicit opioids/substances**	*“The advantages are the patient will decrease their craving for their particular substance, and then possibly stop using their substance of choice.”*	53 (22%)	*“[Helps] stay away from illegal usage”*	52 (22%)	*“Can help to reduce likelihood of use after treatment... No withdrawal to come off medication.”*	38 (16%)	92 (37%)
**Flexible/convenient**	*“Not having. to go to a clinic every day to get dosed and also being able to travel out of town for a period of time because you have your doses with you.”*	11 (4%)	–	0 (0%)	*“It is a once-a-month injection allowing members to have more freedom.”*	57 (24%)	62 (25%)
**Supports recovery lifestyle**	*“The chance to chemically trick your body enabling you the time to change habits, though processes and obtain clean time from the substance you are desperately trying to avoid.”*	26 (11%)	*“This form of [medication assisted treatment] could support their efforts to manage their recovery management and stay away from negative people, places, and illicit activities.”*	37 (16%)	*“Of course this can offer many patients an ability to function and live a productive life.”*	14 (6%)	51 (20%)
**Reduces withdrawal symptoms**	*“It reduces severe opiate withdrawal symptoms, such as perspiration, nausea, anxiety, difficulty sleeping, chills, sensitivity to the light, and stomach pain.”*	28 (11%)	*“Clients can manage their cravings and withdrawal symptoms with methadone use, been used for a long time as a substitute treatment.”*	28 (12%)	*“Activates] to relieve craving and withdrawal it acts as a blocker, preventing other opioids from having any effect.”*	9 (4%)	42 (17%)

**Table 3:** Most prevalent disadvantages and illustrative excerpts

Disadvantages	Buprenorphine (226 responses)	n (%)	Methadone (223 responses)	n (%)	Naltrexone (202 responses)	n (%)	Total (245 responses)
**“No disadvantages”**	-	10 (4%)	-	4 (2%)	-	19 (9%)	24 (10%)
**Creates a long-term dependency**	*“If you stay on it for years, it never gets them off the dependence.”*	45 (19%)	*“Methadone is often referred to as ‘liquid handcuffs’ because clients must travel to and from the clinic daily to receive it and ‘take home doses’ are difficult to earn…”*	47 (20%)	*“A person can become dependent upon it.”*	12 (6%)	74 (30%)
**Side effects**	*“Sedation, nausea, vomiting, itching.”*	35 (15%)	*“Long-term side-effects; damages teeth”*	42 (18%)	*“Nausea, headache, dizziness”; “stomach pain”, “fever”, “allergic reactions”.*	38 (17%)	70 (28%)
**Inconvenient**	*“Having to see the doctor so much because it is so regulated”*	13 (5%)	*“It’s a daily commitment to go to the clinic.”*	68 (29%)	*“You CAN’T [sic] miss that appointment.”*	4 (2%)	68 (27%)
**Specific misuse**	*“[Buprenorphine] is a divertible medication and is frequently diverted as we are learning from our clients. This means they may not use it properly and instead use it to support their ongoing addiction to fentanyl.”*	20 (9%)	*“Patient would use other substances to intensify the effects of the medication to continue use drugs or sell their medication.”*	24 (11%)	*“You can overdose if you try to get high when using it”*	9 (4%)	42 (17%)
**Medication adherence or maintenance difficult**	*“The most important part is keeping up with the prescription and keeping the re-fills.”*	24 (11%)	*“Must be dosed every day and if a dose is missed, then the patient can withdrawal and have a lot of issues. Many patients cannot keep up with it because it does have to be dosed daily.”*	14 (6%)	*“Once [on a] starting regimen many patients don’t keep up with their monthly appointments.”*	16 (8%)	39 (16%)

## Data Availability

The datasets analyzed during the current study are available from the corresponding author on reasonable request.

## Abbreviations

OUD: opioid use disorder
MOUD: medications for opioid use disorder
SUD: substance use disorder
CBH: Community Behavioral Health
